# Recent Advancements in Learning Algorithms for Point Clouds: An Updated Overview

**DOI:** 10.3390/s22041357

**Published:** 2022-02-10

**Authors:** Elena Camuffo, Daniele Mari, Simone Milani

**Affiliations:** Department of Information Engineering, University of Padova, Via Gradenigo 6/A, 35131 Padova, Italy; elena.camuffo@dei.unipd.it (E.C.); daniele.mari@dei.unipd.it (D.M.)

**Keywords:** point cloud, deep learning, compression, scene understanding, semantic segmentation, completion

## Abstract

Recent advancements in self-driving cars, robotics, and remote sensing have widened the range of applications for 3D Point Cloud (PC) data. This data format poses several new issues concerning noise levels, sparsity, and required storage space; as a result, many recent works address PC problems using Deep Learning (DL) solutions thanks to their capability to automatically extract features and achieve high performances. Such evolution has also changed the structure of processing chains and posed new problems to both academic and industrial researchers. The aim of this paper is to provide a comprehensive overview of the latest state-of-the-art DL approaches for the most crucial PC processing operations, i.e., semantic scene understanding, compression, and completion. With respect to the existing reviews, the work proposes a new taxonomical classification of the approaches, taking into account the characteristics of the acquisition set up, the peculiarities of the acquired PC data, the presence of side information (depending on the adopted dataset), the data formatting, and the characteristics of the DL architectures. This organization allows one to better comprehend some final performance comparisons on common test sets and cast a light on the future research trends.

## 1. Introduction

Point clouds have recently appeared among the most promising 3D visual representation models in many heterogeneous fields [1,2,3,4,5,6], ranging from automotive to immersive technologies, such as Augmented and Virtual Reality (AR and VR). Their widespread has been fostered by huge application potentialities such as building safer and smarter autonomous vehicles, preserving endangered cultural heritage sites, implementing smarter video surveillance systems, and enhancing sustainable processes in industrial production, to mention some of them. Moreover, the availability of different sensing devices and algorithms has enabled accurate and detailed acquisitions with diverse computational and development costs. Three-dimensional objects are thus modeled by unordered sets of 3D points sampled over the corresponding surface. Moreover, these representations enable adaptive storage and visualization at different Level-Of-Details (LODs) by simply adding or removing points according to the desired density [7,8,9,10]. Such flexibility derives from the lack of topology and connectivity constraints (as there are in meshes), which makes their acquisition and formatting lighter and better suited for real-time applications.

These advantages come with some structural characteristics that imply significant drawbacks on the processing pipeline and its efficiency, which can be summarized as:

- data sparsity and uneven distribution;
- redundancy of data representation;
- noise and erroneous modeling of object surfaces.

Many point cloud acquisition strategies generate sparse models where points mostly concentrate around key visual features [11]: this leaves large unsampled areas around smooth regions that lead to the presence of holes and missing data. Such sparsity can be utterly accentuated in point-of-view acquisition mechanisms (such as Time-of-Flight or ToF sensors) [12], where the completeness of the model can be impaired by the presence of occlusions and hidden surfaces, and cylindrical acquisition (such as the one adopted by LiDARs), where density varies according to the closeness to the acquiring device [13].

Moreover, point clouds characterize object surfaces by the **spatial repetition of basic elements (points)**: the representation proves to be highly redundant whenever representing planar and regular scenes with respect to a surface-based approach such as with meshes. This results in large models that require a huge storage space or transmission bandwidth [14,15,16,17,18]. As a matter of fact, an accurate data organization is therefore required to handle and visualize the model efficiently (rendering large point clouds turns out to be prohibitive even for a powerful GPU) [19,20,21,22].

Finally, most of the acquisition mechanisms are vision-based, and therefore, they can be highly affected by **external noise sources** such as illumination, object motion, sensor noise, and external radiation sources [23,24,25]. This leads to wrong coordinate estimation [12], together with the appearance of flying pixels and false surfaces [26]. Such impairments can also be found on other sensing devices such as Frequency-Modulated Continuous Wave (FMCW) radar [25], where environmental factors can deeply affect the quality of the resulting acquisition and the final processing performance [27,28].

For these reasons, several research efforts have been recently dedicated to the development of effective point cloud processing solutions, together with the ability of understanding and interpreting the acquired scene, including interpolation and completion algorithms, compression mechanisms, data organization schemes, classification [29,30], and segmentation strategies [6].

During the last ten years, deep learning solutions have seen a huge technical development thanks to the availability of high-performing GPUs and large training datasets. Such inherence has led to the creation of accurate and performing point cloud processing algorithms that have overcome the performance of previous traditional computer vision solutions [31]. On the other hand, the heterogeneous characteristics of point cloud data, as well as the data-oriented nature of DL solutions, have brought forward some issues and opened new research problems. From these premises, the analysis of the panorama of PC processing solutions needs to be revisited and reorganized according to new classification criteria.

The main aim of this work is to present an updated and reorganized overview of the latest PC solutions where the different approaches are analyzed in light of a new taxonomical classification that was derived from the fundamental features of DL technologies. Most of the previous works and overviews divide the analyzed strategies according to the neural architecture (block structure, layer types, etc.) or their specific neural learning categories (Convolutional Neural Networks, Generative Adversarial Networks, etc.). In our analysis, we have seen that it is not possible to disregard other peculiarities such as the acquisition method or sensors, the sparsity of the points, the organization of the data processed by the networks, and the characteristics of the datasets. These details can be schematized as follows.

- **Data organization and formatting**: point cloud data can be stored and sampled in different ways depending on the acquisition strategy and on the following operations. Point coordinates can be quantized, fused together, sampled, or erased in order to simplify or reduce the noise level and improve the subsequent processing steps.
- **Acquisition strategy and sensor integration**: point cloud data can have very diverse statistics depending on the algorithm or device that was used to generate it. As a matter of fact, LiDAR and Structure-from-Motion PCs can be much sparser than ToF-generated models. This introduces further distinctions between the different network architectures that can be tailored and optimized for a specific type of data.
- **Network/layer type and architecture**: specific NN architectures could be more suitable for a given task; therefore, in the last years, a significant research effort has been dedicated to the investigation of the most effective network structures, as well as of new processing layers that prove to be more efficient in targeting the characteristics of the input. As a result, a few architectures have been widely reused for specific tasks such as classification, coding, or semantic segmentation.
- **Training/Learning strategy and adopted loss**: recent developments in neural networks have shown that the learning paradigm can lead to very different performances with the same dataset and network scheme. This evidence has led to the development of different paradigms such as curriculum, continual, and contrastive learning, to mention a few. Aside from the learning paradigm, loss functions have shown a crucial role in the convergence speed, as well as in the final accuracy.

**Figure 1:**
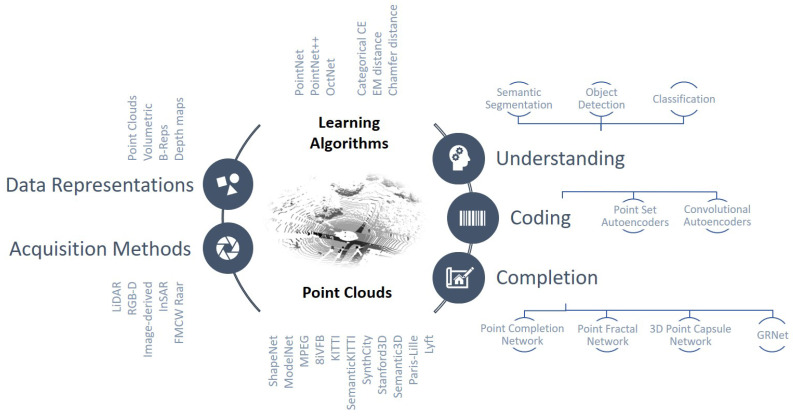
Schematic representation of the paper organization.

This paper overviews the most recent NN-based processing strategies for point clouds, starting from simple classification problems and ending with the most advanced semantic interpretation mechanisms (see Figure 1). The description presents the different strategies classified according to their characteristics among the aforementioned, which are detailed in the following sections (see Table 1).

Section 2 presents the different data formats that are employed by different network structures, while Section 3 overviews the main datasets and acquisition mechanisms that are currently available and used in the literature. Section 4 describes the most widely used network models that constitute the core building blocks for most of the processing architectures and the adopted loss functions. Section 5 deals with the problem of semantic scene understanding, while Section 6 presents the main learned compression schemes. Section 7 describes the deep-learning completion strategies while the final conclusions are drawn in Section 8.

## 2. Point Clouds as Data Structures

The latest 3D acquisition mechanisms have enabled the modeling of real 3D scenes by means of unordered sets of 3D points, which can be accompanied by different attributes including color components, normals, semantic labels, and sensing-related measurements (such as reflectance in LiDAR acquisitions). In comparison to standard 2D images, point clouds require an increment of the storage space (as each file consists of both geometry and attribute information), as well as of the processing computational load. As a matter of fact, different data structures have been proposed in order to enable efficient handling of the acquired data.

### 2.1. Point Cloud Data

**Figure 2:**
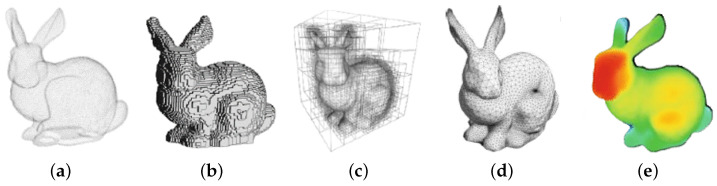
The Stanford Bunny [38] model in different three-dimensional representations. (**a**) Point Cloud, (**b**) Voxels, (**c**) Octree, (**d**) Mesh, (**e**) Depth.

The most straightforward formatting strategy is through a list of three-dimensional points sampled over the surface of the objects since this representation is the closest one to the raw sensor data format. Specifically, a point cloud (see Figure 2a) is a set of three-dimensional coordinate data [32], where each point is spatially defined by a triplet of coordinates, e.g., (x,y,z). A dense set of 3D points can efficiently model the surface of an object or a complete scene, which can be enriched by additional point features, related to the specific acquisitions or generation strategies. The most common real-time acquisition methods are the optical 3D sensing devices such as LiDAR or ToF sensors. However, PCs can also be the result of photogrammetric scanning, multiview reconstruction, RADAR estimation, and deep generative methods such as those employing Generative Adversarial Networks (GANs). Recently, synthetic point cloud datasets from simulation environments have also become very popular. Point clouds provide simple yet efficient and precise representation and can be subjected to operations such as fast linear transformations, objects combinations, and fast rendering. These benefits, on the other hand, must contend with memory constraints and free space representation issues. Since the same surface or position is sensed several times, this results in multiple overlapping dense points; moreover, nonmeasured space (because of noise or lack of visual features to be used in the reconstruction) is treated similarly as free space. Finally, even if fast rendering can be applied directly on 3D points, a solid representation of object geometries is usually more efficient, and therefore, mesh reconstruction algorithms are often applied with a significant computational effort.

### 2.2. Acquisition Systems

Data and algorithm selections are strongly driven by the requirements of specific applications. As a result, it is possible to distinguish different types of point cloud data, depending on the technologies used for the acquisition/generation.

- **Light Detection And Ranging (LiDAR)** are detection systems that resemble the operation of radar but, instead, use the light from a laser, producing a sparse prediction of the environment in the form of point clouds. These sensors are increasingly being applied in multiple fields, such as robotics, mobile mapping, and autonomous driving, and they can provide large-scale datasets with more than one million points, either in static [33] or dynamic [34,35] environments.
- An **RGB-D camera** is a type of sensor that can acquire both RGB and depth information. RGB-D sensors are usually applied to capture point cloud data in an indoor environment, because of their range limitations.
- **Image-derived methods** generate a point cloud indirectly from stereo or multiview images. Image-derived point clouds have been frequently used in real-world scenarios; however, there are not many studies on image-based data.
- **Interferometric Synthetic Aperture Radar (InSAR)** is a radar technique for remote sensing. It generates maps of surface deformation or digital elevation based on the comparison of two or more SAR images. InSAR-based point clouds are creating new possibilities for point cloud applications, even though there are not many studies yet.
- **Frequency-Modulated Continuous Wave Radar (FMCW Radar)** is a special type of radar sensor which radiates continuous transmission power such as a simple continuous wave radar. FMCW radar has the peculiarity that it can change its operating frequency during the measurement, i.e., the transmission signal is modulated in frequency (or in phase). FMCW radars offer robust sensing to autonomous vehicles [27,36], for their high-range resolution and accuracy: in fact, FMCW radars add an extra dimension in the sensing, and they are more robust to weather changes, with respect to LiDAR sensors. FMCW radars are often employed also in Human Motion Detection [25,37] and Activity Recognition systems [28].

### 2.3. Other Data Structures

When dealing with deep learning algorithms, point clouds are usually not the most suitable data structure to process. Thus, the input data are frequently subject to transformations that allow them to satisfy the specific needs of the architecture. Among other data structures, we can find volumetric models, shell or boundary models, parametric models, and depth maps.

#### 2.3.1. Volumetric Models

Volumetric models are the most common and intuitive representation of three-dimensional data as they are just an extension of bidimensional images to the third dimension [4]. We can consider **voxels** (see Figure 2b) as the equivalent three-dimensional representation of a pixel from a bilevel image. A voxel is formally a three-dimensional cubic unit block that represents a naive extension of occupancy grids to a 3D space (an occupancy grid is a bidimensional space, representing an environment, subdivided through a grid system where occupied cells are filled while those relative to free space are not). A sparse voxelization can be obtained directly from point clouds, by discretizing the space and filling voxels where one or more points are present. Unfortunately, this representation results are quite rough and bulky, and hence, data can be organized more efficiently by means of octree structures. **Octrees** (see Figure 2c) are tree-based data structures that progressively refine the representation of 3D space by recursively partitioning the occupancy volume into octants and keeping track of the nonempty (occupied) subregions. Volumetric representations are practical for rendering and smooth visualization. However, they perform a rough approximation of the initial geometry and introduce significant aliasing artifacts whenever voxel resolution is not high enough. As a matter of fact, they are mostly used in three-dimensional convolutional neural architectures (thanks to their highly structured grid layout).

#### 2.3.2. Shell or Boundary Models

Shell or boundary (B-Reps) models are usually employed to represent the boundaries or surfaces of the objects. Almost all visual models used in reality capture workflows, games, and movies are boundary representations. Among them, **triangular meshes** (see Figure 2d) are the most commonly used in computer graphics. A mesh is a geometric data structure that encodes a three-dimensional object geometry in terms of a combination of edges, vertices, and faces. Meshes are a great way to explicit the geometry of a point cloud, and they frequently allow for a significant reduction in the number of points required as vertices. On top of that, they permit one to obtain a sense of the relationship between objects through the faces’ connectivity. However, meshing is an interpolation of the base point cloud geometry and can only represent the data to a certain degree, which is determined by the mesh’s complexity. There are a variety of ways for meshing a point cloud, but the best results typically necessitate some prior knowledge of the object’s shape.

#### 2.3.3. Depth Maps

Depth maps (see Figure 2e) are images that encode depth information of a three-dimensional scene from a single viewpoint. This information is encoded using height above the ground, horizontal disparity, and angle with gravity for each pixel. This representation is accurate and dense if the surface radiated by the sensor or associated with a visual feature is consistent (i.e., associated with a uniform depth measurement) and wide enough. For example, in real-time autonomous driving scenarios, depth maps allow one to map the environment at $360^{\circ }$ in real-time. A depth map is a good data structure for its low memory requirements, but it suffers from weak topology and cannot generate a full three-dimensional description of the scene without fusing multiple diverse viewpoints.

## 3. Datasets

In this section, we briefly present some of the most popular point cloud and 3D sample datasets that have been proposed in association with different deep learning tasks, ranging from compression to semantic segmentation and classification. Such repositories currently represent the main state-of-the-art benchmarks on which most algorithms are evaluated and compared.

### 3.1. ShapeNet

**Figure 3:**
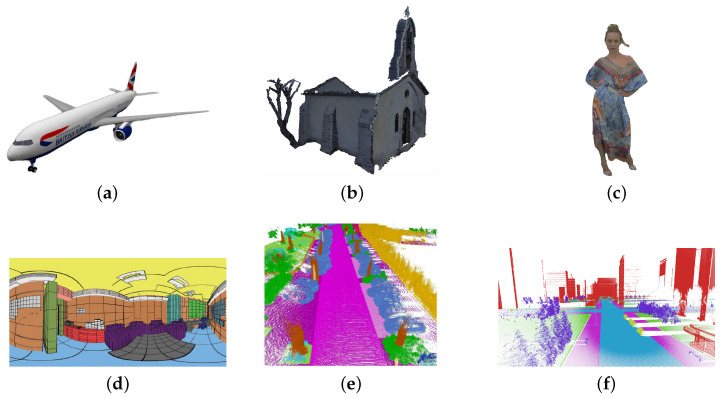
Samples from some of the presented datasets. (**a**) ShapeNet, (**b**) MPEG, (**c**) 8iVFB, (**d**) S3DIS, (**e**) SemanticKITTI, (**f**) SynthCity.

ShapeNet [39] is a very large repository of shapes (see Figure 3a) comprehending many different semantic classes organized under the WordNet [40] taxonomy. Those were gathered from various 3D CAD model repositories and were labeled so that the resulting trained models can solve different tasks. The annotations assigned to the data samples can be divided as follows:

- **Language-related annotations**: category labels are assigned to the objects according to the WordNet taxonomy; this labeling is helpful for indexing tasks and can be used to train-shape classification models.
- **Geometric annotations**: information about the object orientation, symmetry planes, and scale are provided; in general, labels also identify the main object parts that compose each model.
- **Physical annotations**: physical properties of the surface materials and weights are also provided to enable physical simulations.

The large number of labels that are provided in this dataset, make it a perfect candidate for many different tasks such as classification, part segmentation, data generation, and reconstruction. The main issue with this data is that since they are synthetic they produce perfect PCs far from the noisy and incomplete ones obtained by sensors.

### 3.2. ModelNet

Similar to ShapeNet, ModelNet [41] is a repository of CAD 3D models that can be divided into 40 classes of object shapes. Also in this dataset, the models are synthetically generated and therefore, although artifact-free, they are not very faithful with respect to real data.

Most of the time, these two datasets are used in conjunction using Shapenet as training data and ModelNet as testing models.

### 3.3. MPEG

MPEG test conditions [42] include a set of reference point clouds that have been acquired with different strategies spanning from Structure-from-Motion to ToF sensors to LiDAR data (see Figure 3b). More precisely, dynamic sequences were acquired with ToF and LiDAR sensors, while static data include models from laser scanners and sampled multicamera reconstruction as well. Models are organized into three categories including static models, dynamic objects, and dynamic acquisitions. Data were generated from multiple repositories by several contributors including Microsoft, CERTH, 8i, Queen Mary University, IMT, and UPM, to mention some of them.

### 3.4. 8i Voxelized Full Bodies

The 8i Voxelized Full Bodies (8iVFB) consists of four point cloud sequences (longdress, loot, redandblack, and soldier), where the full body of a human subject (see Figure 3c) is captured by 42 RGB cameras configured in 14 clusters (each cluster acting as a logical RGB-D camera), at 30 fps, over a 10 s period [43]. Spatial coordinates are quantized with 10 bit resolutions representing each model with a 1024×1024×1024 voxel cube; for each sequence, the cube is scaled so that it is the smallest bounding cube that contains the entire sequence. The dataset represents the reference data for the JPEG Pleno Dataset.

### 3.5. Stanford 3D Indoor Scene Dataset

The Stanford 3D Indoor Scene Dataset (S3DIS) [44] contains 6 large-scale indoor areas with 27 rooms (see Figure 3d). Each point in the scenes is annotated with one of the 13 semantic categories. S3DIS belongs to the category of static datasets [45], which are commonly used for point cloud classification tasks, but it is designed to be suitable even for Semantic Segmentation, Instance Segmentation, and Object Detection tasks. Its main application scenarios include robotics, augmented reality, and urban planning.

### 3.6. KITTI

KITTI [34] is one of the most popular publicly available datasets for autonomous driving. It is a sequential dataset, acquired with an autonomous driving system to capture the sequences of LiDAR frames with a moving viewpoint on the street. The system is composed of a LiDAR sensor, a stereo camera rig (RGB-D), a Global Positioning System (GPS), and Inertial Measurement Unit (IMU) to allow different tasks of interest: stereo, optical flow, visual odometry, 3D object detection, and 3D tracking.

As a sequential dataset, KITTI contains several frames but sparse points. Furthermore, since sensors’ viewpoints follow the direction of the roads (the acquisition equipment was installed on a vehicle), the sampled LiDAR points associated with the road label are distributed at specific angles, which can be easily predicted from the knowledge of the system’s settings.

### 3.7. SemanticKITTI

SemanticKITTI dataset [35] was built on the velodyne sequences of KITTI dataset and provides in addition to 3D data, the Semantic Segmentation and Panoptic Segmentation labels (see Figure 3e). It contains detailed point-wise annotations with 28 classes, on 22 different scenes, and it is one of the biggest public datasets for autonomous driving.

### 3.8. SynthCity

SynthCity [46] is a 367.9 M point clouds dataset acquired with Mobile Laser Scanning in a simulated environment (see Figure 3f). Every point is assigned a label from one of 9 categories. The problem of using these kinds of datasets is the large gap between synthetic and real scenes. The former can generally be very realistic, but they lack accuracy in detail, even if they are extremely easy to label and acquire.

### 3.9. Other Recent LiDAR Datasets for Automotive Applications

Semantic3D [33] is the existing largest LiDAR dataset for outdoor scene segmentation tasks with more than 4 billion points. Paris-Lille-3D [47] is smaller than Semantic3D, with more than 140 million labeled points. It was acquired with a mobile LiDAR in two French cities, Paris and Lille, and well suits autonomous vehicles’ applications. Finally, Lyft [48] is a novel proposed dataset for the perception of urban scenarios, and it is coupled with another dataset for the prediction of vehicles’ trajectories. It includes more than 30,000 LiDAR point clouds with 1.3 million annotations.

## 4. General Purpose Deep Learning Techniques

In this section, we provide a brief overview of some state-of-the-art NN architectures and losses, which are employed in multiple PC-related domains. The subsequent sections analyze how these are employed in the different PC processing steps.

### 4.1. Architectures

The architectures used for point cloud processing are generally designed to accomplish more than one deep learning task. Some of these directly process point coordinates, while others work on different data structures.

#### 4.1.1. PointNet

One of the most successful architectures is PointNet [49], which was proposed both for classification and segmentation purposes. Its main feature is that it directly processes point spatial coordinates instead of voxel grids, as other same-purpose schemes do. This solves some sparsity problems since the latter usually implies characterizing a lot of empty subregions; as a result, voxel-based solutions require larger storage space, as well as a lot of useless computations (e.g., in 3D CNNs architectures). The main drawback of this model is that it does not take into consideration the local correlation between neighboring points: this effect proves to be crucial in a correct classification as it is nicely investigated in [29].

Since it processes an unordered set of coordinates, PointNet is designed to be permutation invariant. This property is attained by computing point-wise features that are then merged into a single vector by applying a transformation *h* followed by a symmetric function *g* (e.g., the sum or the multiplication):

(1) $$f\left(x_{1},\cdots ,x_{n}\right)=g\left(h\left(x_{1}\right),\cdots ,h\left(x_{n}\right)\right).$$

This is repeated for multiple *g*, *h* functions to learn different properties of the data.

In order to enable scale, rotation, and translation invariance, an affine registration transform is learned in order to standardize the data points.

**Figure 4:**
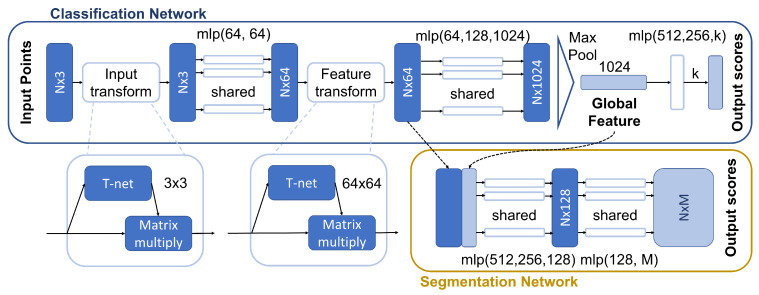
PointNet [49] model.

A block diagram of the architecture can be seen in Figure 4. It is possible to notice that the first block is the T-net unit. This block takes as an input tensors $T\in R^{b\times n\times 3}$, where *b* is the batch size and *n* is the number of points; the output is a tensor of the same size that is standardized by multiplying *T* by the learned affine transform. In detail, the sequence of operations can be summarized as follows:

- expand dim: $R^{b\times n\times 3}⟶R^{b\times n\times 3\times 1}$
- 2D convolution with 64 kernels with size [1,3], no padding, stride 1, ReLU and BatchNorm: $R^{b\times n\times 3\times 1}⟶R^{b\times n\times 1\times 64}$
- 2D convolution with 128 kernels of size 1, no padding, stride 1, ReLU and BatchNorm: $R^{b\times n\times 1\times 64}⟶R^{b\times n\times 1\times 128}$
- 2D convolution with 1024 kernels of size 1, no padding, stride 1, ReLU and BatchNorm: $R^{b\times n\times 1\times 128}⟶R^{b\times n\times 1\times 1024}$
- Max pooling with size [n,1]: $R^{b\times n\times 1\times 1024}⟶R^{b\times 1\times 1024}$
- fully connected layers to obtain an affine transform for each point cloud in the batch: $R^{b\times 1\times 1024}⟶R^{b\times 3\times 3}$

This sequence is repeated on the point cloud features. The function *h* actually consists of the following pipeline: $T_{Net}⟶MLP⟶T_{Net}⟶MLP$; on the other hand, the symmetric function *g* is a max pooling over all points. It is important to notice that, in the MLPs, weights are reused for each feature vector associated with the point (this operation is essentially a 1D convolution). Thanks to the global max pooling and the convolution operations, it is actually possible to feed a variable number of points to the network and the algorithm will still yield 1024 global features.

Since the transformation matrix for the 64-dimensional features is quite big and hard to learn, the authors add a regularization component $L_{reg}$ to the loss that forces the transformation matrix to be close to an orthogonal one:

(2) $$L_{reg}=||I-AA^{T}{\left|\right|}_{F}^{2}$$

#### 4.1.2. PointNet++

As previously mentioned, PointNet does not exploit local correlation in the data. In order to mitigate this lack of data and improve the efficiency of the architecture, PointNet++ [50] was introduced: the enhanced scheme divides the PC into smaller sets of neighboring points where features are extracted and merged recursively.

Although it solves the issue of local correlation, this technique introduces the need to define an efficient partitioning strategy to create the PC subsets. The authors decided to choose the centroids with the furthest point sampling algorithm that in general has better data coverage w.r.t. random sampling. In order to find the groups of points, ball queries or KNN can be used.

This allows a hierarchical merging of PointNet features from local clusters characterizing the local statistics and processing the input point set in a bottom-up manner [29].

#### 4.1.3. Convolutional Neural Networks

Differently from per-point feature extractors such as PointNet and PointNet++, local correlation in PC processing can be exploited by means of Convolutional Neural Networks (CNNs). Such architectures have already been thoroughly tested in 2D signal processing and a wide variety of architectures have already been proposed to solve various tasks. As a matter of fact, their extension to the 3D case has been a natural implication [30]. An effective application of 3D convolution implies representing the input PC as a voxel grid which implies the quantization of point coordinates (introducing an additional quantization noise component on geometry data) and the characterization of many empty spaces (generating a lot of useless operations). For these reasons, the computational complexity of CNNs scales cubically with the grid resolution.

#### 4.1.4. OctNet

One approach that tries to tackle the computational complexity issue arisen by CNNs is OctNet by Riegler et al. [51], where the authors propose a solution based on octrees that reduces the complexity of the problem. An octree is a data structure used to represent a voxel volume, allowing one to model densely populated regions with high precision while empty regions are summarized by a big empty cell. This optimizes the representation of the voxel volume, but it has the problem that accessing a random element turns out to be very slow depending on its depth in the coding tree. This drawback may be critical for real-time PC processing. For this reason, the authors proposed the Hybrid Grid-Octree, where multiple “shallow” octrees with maximum depth equal to 3 are distributed in a grid within the voxel volume. As a result, whenever large areas of the volume are actually empty, octree coding represents them with a long sequences of zeros on which convolution operations can be skipped, thus reducing the overall computational complexity. This allows this architecture to process point clouds at a much higher resolution.

### 4.2. Losses

In order to properly optimize a neural network, a cost function that is to be minimized needs to be defined. When considering PC data, there are some loss expressions that are recurrently used in multiple tasks; a brief introduction to the most popular ones is provided below.

1. **Categorical Crossentropy**
  Categorical Crossentropy is the most common loss choice for DL tasks that involve the assignment of different classes to the samples (e.g., classification and semantic segmentation). In this case, the model should output for each sample $x_{i}$ a probability distribution vector ${\hat{y}}_{i}$ (easily obtainable with a softmax activation function) where each entry ${\hat{y}}_{i,j}$ represents the probability that sample $x_{i}$ belongs to class $c_{j}$. In particular for the multiclass classification task given the predicted classes and the ground truth, respectively $\hat{y},y\in {\left[0,1\right]}^{n\times c}$, where *n* is the number of samples and *c* the number of classes, the categorical crossentropy loss is defined as:
  (3) $$CE\left(y,\hat{y}\right)=-\frac{1}{n}\sum_{i=1}^{n}\sum_{j=1}^{c}y_{i,j}log\left({\hat{y}}_{i,j}\right)$$
2. **Earth Mover’s Distance**
  The Earth Mover’s Distance (EMD) [52] is a permutation invariant metric that computes the minimum movement required for point set $S_{1}$ to be mapped into point set $S_{2}$. Considering the two sets need to have the same cardinality, it is possible to write it as:
  (4) $$d_{EMD}\left(S_{1},S_{2}\right)=\min_{\phi :S_{1}\to S_{2}}\sum_{x\in S_{1}}||x-\phi \left(x\right){\left|\right|}_{2}.$$
  The main drawbacks of this metric are the computational demand and the lack of differentiability; as a matter of fact, its use in training operations is not easy, and therefore, it is commonly used for evaluation in PC compression, interpolation, completion, and generation.
3. **Chamfer (Pseudo) Distance**
  An alternative to EMD is the Chamfer (pseudo) Distance (CD), which measures the squared distance between one element in $S_{1}$ with the nearest neighbor in $S_{2}$ and vice versa. It can be expressed as:
  (5) $$CD\left(S_{1},S_{2}\right)=\sum_{x\in S_{1}}\min_{y\in S_{2}}||x-{y||}_{2}^{2}+\sum_{y\in S_{2}}\min_{x\in S_{1}}||y-{x||}_{2}^{2}.$$
  The main advantages of this metric are that it does not require $|S_{1}\left|=\right|S_{2}|$, it is differentiable everywhere, and it is actually faster for computing w.r.t. EMD. As a consequence, it is usually the best choice when considering point prediction tasks that do not rely on a voxel grid (completion, generation, interpolation, and compression).

## 5. Semantic Scene Understanding

Semantic scene understanding is an essential computer vision task in many application fields (such as autonomous driving, remote sensing, and robotics), and the new possibilities offered by deep learning techniques have inspired many research efforts in this direction. The main aim is to identify and classify each element within an acquired scene, i.e., analyze the objects starting from the three-dimensional coordinates of the acquired samples, their layout, and their spatial, functional, and semantic mutual relationships.

### 5.1. Disambiguation

Semantic scene understanding includes several computer vision tasks that provide understanding at different levels. Among these, we can mention:

- **Classification:** identifies the content of a point cloud and categorizes it with a single geeral label. Formally, given a set of points, $X=\{x_{1},x_{2},\cdots ,x_{N}\}$ and a candidate label set $Y=\{y_{1},y_{2},\cdots ,y_{k}\}$ assign the whole point set *X* to only one of the *k* labels.
- **Object Detection:** localizes all the objects in the scene and encapsulates each of them into a bounding box; in this way, the task estimates the geometric location and orientation in addition to semantic instance labels. Each box is commonly represented as (x,y,h,w,θ,c). The parameters (x,y) denote the object (bounding box) center position, while (h,w) represents the bounding box size with width and height, and θ is the object orientation. Finally, *c* represents the semantic label of this bounding box (object).
- **Semantic Segmentation:** clusters the input data into several homogeneous regions, where points in the same region have identical attributes. Each input point can be associated with a semantic label, i.e., given a set of *N* ordered points $X=\{x_{1},x_{2},\cdots ,x_{N}\}$ and a candidate labels set $Y=\{y_{1},y_{2},\cdots ,y_{N}\}$, assigned to each $x_{i}$ one label $y_{i}$. If the instances of a category of objects are recognized as different entities, the task is named *Instance Segmentation*, while if we are referring to a differentiation among the parts of a single point cloud, we are referring *Part Segmentation*.

**Figure 5:**
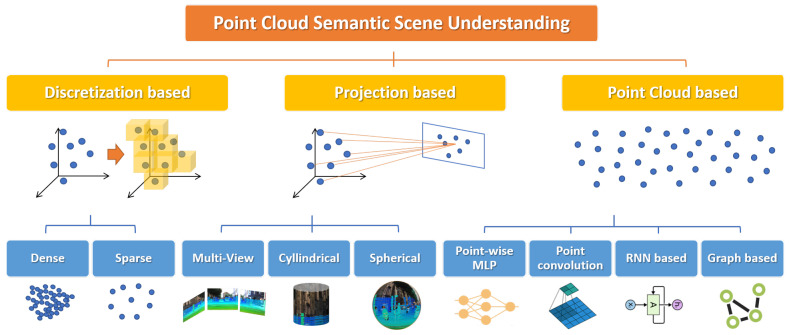
Taxonomy of the main methods for PC Semantic Scene Understanding.

The main architectures and methods in PC semantic scene understanding can be categorized according to the format of input point cloud data (Figure 5). Hereinafter, we overview the most successful deep learning models, with the main focus on PC Semantic Segmentation, but considering also Object Detection and Classification, since the most popular architectures generally address more than one task.

### 5.2. Discretization-Based Models

Discretization-based methods transform point clouds into discrete data structures before feeding them to the network architecture. These structures can be dense, such as voxels or octrees, or sparse, such as permutohedral lattices (in mathematics, the permutohedron of order *n* is an (n−1)-dimensional polytope embedded in an *n*-dimensional space). Their main advantage is that these structures can be treated as three-dimensional images and dense or sparse convolutions can be easily applied.

#### 5.2.1. Dense

The idea behind these methods is to divide the space occupied by point clouds into volumetric occupancy grids (voxels or octrees) and assign the same label to all the points belonging to the same cell. Then, using a convolutional architecture, as Huang et al. in [53], a prediction is computed for each voxel center and assigned to the neighboring points.

**Figure 6:**
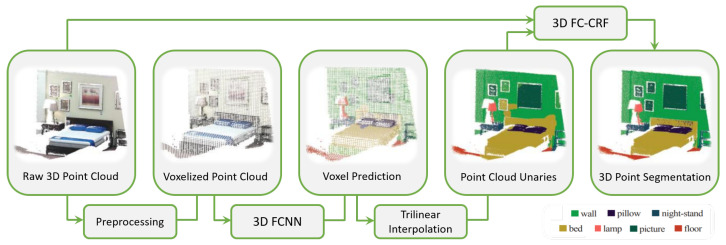
SegCloud [54] pipeline.

The advantage of using these data structures is that both the three-dimensional shape and viewpoint can be encoded and voxels can be classified according to the particular condition in occluded, self-occluded, and visible voxels. Nevertheless, the performance is severely limited by voxel density and boundary artifacts caused by the point cloud partition. SEGCloud by Tchapmi et al. [54] brought an improvement introducing fine-graining and global consistency; this result was achieved by using trilinear interpolation to remap predictions to point cloud and Fully Connected Conditional Random Fields (CRFs) to enforce spatial consistency of predictions (Figure 6).

3D ShapeNets [41] was proposed by Wu et al. jointly with the ModelNet dataset (Section 3) to solve the classification task. It employs a Convolutional Deep Belief Network (CDBN) to describe the geometric shape of a 3D voxel grid as a probability distribution of binary variables. A CDBN is a type of deep artificial neural network composed of multiple blocks of convolutional restricted Boltzmann machines stacked together, which is translation invariant and scales well to high-dimensional images. This model automatically learns the hierarchical compositional part representations of 3D objects, and it can also be optimized for completion purposes (Section 7). Although it achieves impressive results with low-resolution voxel grids, the performance of the model is limited. VoxNet [55] was proposed by Maturana et al. for 3D object recognition, and using 3D convolution filters is another pioneer in volumetric data processing. From these initial works, 3D convolution has been widely adopted showing a good accuracy even in challenging acquisition set-ups [30]. A different method using an adversarial scenario is proposed in 3D GAN [56], which is a volumetric CNN composed of a generator and a discriminator, both made of five volumetric fully convolutional layers. The generative-adversarial criterion has the advantage in capturing the structural variation between two 3D objects, but its limitation can be seen in memory occupation and in low 3D resolution.

OctNet [51] (already described in Section 4) better exploits the sparsity of the input data and permits improving the performances of voxel-based methods. This approach hierarchically partitions the space with a series of unbalanced octrees, which adapt the memory allocation and computation according to the point density of different volumetric regions.

#### 5.2.2. Sparse

As already stated before, the inherent sparsity of many point cloud models makes the percentage of occupied cells in volumetric representations quite small. For these reasons, sparse convolutional networks could be more efficient, as was highlighted by many approaches in the literature. Graham et al. [57] proposed Submanifold Sparse Convolutional Networks, a technology that efficiently minimizes memory usage and computational complexity, suited for high-dimensional and spatially sparse data.

Important contributes are given by Su et al. with SplatNet [58] and by Rosu et al. with LatticeNet [59]. LatticeNet, developed after SplatNet, is based on Permothoedral Lattices and computes Sparse Convolution (SC) with slight modifications, achieving efficient processing of large-scale point clouds.

### 5.3. Projection-Based Models

Projection-based models use a bidimensional projection of point clouds to infer predictions, i.e., they remap the input data to a simpler and easier to handle structure. These methods are based either on multiview, spherical, or cylindrical projections. The considered deep learning architectures are usually well-established convolutional neural networks (CNN) models (eventually pre-trained on image datasets), such as AlexNet [61], VGG [62], GoogLeNet [63], ResNet [64]. Compared with discretization-based models, these methods can improve the performance for different 3D tasks by taking multiple views of the objects or scenes of interest and then fusing the outputs or performing majority voting to produce the final prediction; additionally, they are efficient in terms of computational complexity. On the other hand, this strategy implies an information loss, and its performances highly vary depending on the projection viewpoints or directions.

#### 5.3.1. Multiview

Multiview CNN (MVCNN) by Su et al. [65] was one of the pioneering 2D DL models in 3D estimation via the merging of different multiview features (generated by means of different MaxPooling stages) into a global descriptor. However, this operation retains the most important features only and fails to preserve comprehensive visual information. MVCNN-MultiRes was proposed by Qi et al. [66] to improve MVCNN by introducing multiresolution 3D filtering to capture multiscale information. Nevertheless, multiview methods cause numerous limitations and a loss in geometric structures. To tackle this problem, approaches such as SnapNet [67] apply CNNs on multiple 2D image views generating one RGB map and one depth map to preserve geometric features; labels are assigned to both images before reprojecting back to 3D space.

#### 5.3.2. Spherical

An efficient way to obtain fast and accurate PC segmentation was proposed by Wu et al. with SqueezeSeg [68], an end-to-end network based on CRFs and SqueezeNet [69]. SqueezeSegV2 [70] was then introduced to address domain shift, i.e., including also synthetic data in the training procedure, by utilizing an Unsupervised Domain Adaptation (UDA) pipeline. Milioto et al. proposed RangeNet++ [71] for real-time semantic segmentation of LiDAR point clouds.

#### 5.3.3. Cylindrical

Cylindrical coordinate systems have recently proved to be very effective in LiDAR PC representation for different tasks. PolarNet [72] instead of common spherical or bird’s-eye-view projection, balances the points across grid cells in a polar coordinate system, indirectly aligning a segmentation network’s attention with the long-tailed distribution of the points along the radial axis.

### 5.4. Point Clouds-Based Models

To avoid limitations posed by both projection and discretization based methods, many approaches resort to processing point cloud data directly.

#### 5.4.1. Pointwise MLP Methods

This class of methods have been introduced by Qi et al. in 2017 with PointNet [49] (explained in detail in Section 4). This is one of the pioneering frameworks in PC semantic segmentation as it achieved considerable results avoiding the use of convolutional networks, learning per-point features using shared MLPs and global features using symmetrical pooling functions. Following the PointNet trend, other works have adopted a shared MLP as the basic processing unit thanks to its high efficiency. PointNet++ [50] (presented in Section 4) was introduced to capture local structures, introducing a hierarchical network that applies PointNet recursively, learning local features with a progressively increasing contextual scale based on K-nearest-neighbor (KNN) and query-ball searching methods.

**Figure 7:**
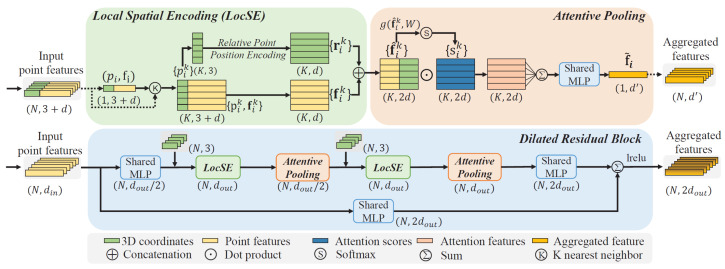
RandLA-Net [60] basic module.

A pioneer work for large-scale point cloud segmentation is represented by RandLA-Net [60]. In this work, Hu et al. proposed an efficient and lightweight network that captures context information of each point neighborhood and employs an effective local feature aggregation module (Figure 7), which automatically preserves complex local structures by progressively increasing the receptive field. Furthermore, this network only relies on random point sampling to achieve remarkably high efficiency in terms of memory and computation.

#### 5.4.2. Point Convolution Methods

**Figure 8:**
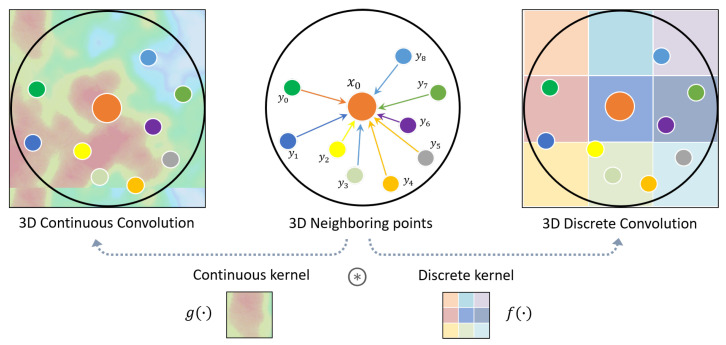
Different types of point convolution [32].

These methods adopt specific 3D convolution operators tailored on point clouds, which can be either continuous or discrete: **3D Continuous Convolution** kernels are defined on a continuous space, where the weights for neighboring points are related to the spatial distribution with respect to the center point. **3D Discrete Convolution** kernels are defined on regular grids, where the weights for neighboring points are related to the offsets with respect to the center point. (Figure 8).

PointConv [73] firstly introduced novel continuous convolution kernels as nonlinear functions and proposed a formulation for efficiently scaling up the network and improving its performance, while KPConv [74] proposed a deformable version of this convolution operator that learns local shifts effectively deforming the kernels to fit them to the point cloud geometry. Similarly, (AF)${}^{2}$-S3Net [75] fuses voxel-based and point-based learning methods into a unified framework to effectively process large 3D scenes.

**Figure 9:**
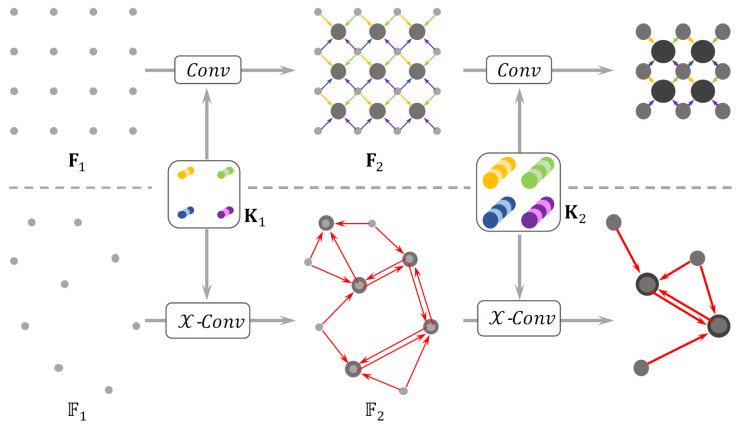
PointCNN [76] χ-Conv operator.

Among discrete convolution-based approaches are neural architectures that employ simple CNN layers for classification and localization tasks [37]. The pioneer method PointCNN [76] introduces a χ-Conv operator (Figure 9) that weights and permutes input points and features before they are processed by a typical convolution. This allows one to canonicalize point order and learn generalized convolutional features from unordered and unstructured point clouds. On the other hand, the introduction of skip connections, as in [36], has proven to boost the performance of convolutional networks: a UNet-like architecture is adopted in this work for classification and object detection in mmWave-radar point clouds.

**Figure 10:**
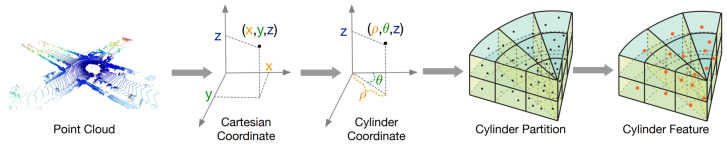
Cylinder3D [77] space partition.

More recently, Cylinder3D [77] (Figure 10) was introduced in the context of automotive point clouds for driving-scene modeling. This architecture exploits the 3D topology relations and structures of sparse point clouds to build a cylindrical volumetric partition and explore context information in an effective progressive manner.

**Figure 11:**
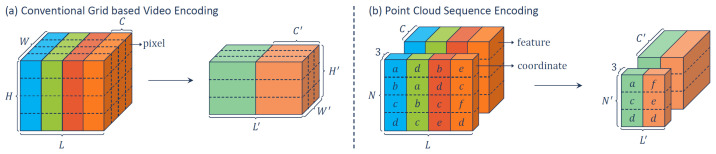
PSTNet [78] sequence encoding.

Finally, approaches have been developed to embed the temporal dimension in the processing. Fan et al. proposed PSTNet [78] (see Figure 11), a deep network that hierarchically captures features from a Point Spatiotemporal (PST) convolution. PST combines a spatial convolution to capture the local structure of points in the 3D space and a temporal convolution to model the dynamics of the spatial regions along the time dimension.

#### 5.4.3. RNN-Based Methods

RNN-based methods have been introduced to model the interdependency between point cloud acquisitions at different subsequent time instants (usually called *frames* in analogy with standard 2D video). One of the major works exploiting this idea is PointRNN [79] which proposes the network in two different versions depending on the recurrent module introduced, i.e., PointGRU and PointLSTM. Other solutions combine the efficiency of CNN with the recurrent architectures such as in the classification approach by Pirasteh et al. [6].

### 5.5. Graph-Based Methods

**Figure 12:**
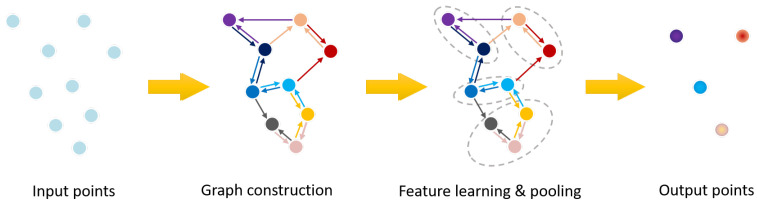
Graph-based networks principle [32].

Finally, several methods resort to networks that process graphs (see Figure 12), which are really suitable structures to represent point clouds as they can capture the geometric structure and shape of objects. When representing point clouds with graphs, each node corresponds to an input point and each edge represents the relationship between the point and its neighbors.

Landrieu et al. [80] used superpoint-graph to capture the structure and context information of large-scale point clouds. The segmentation problem is split into three subproblems, i.e., geometrically homogeneous partition, superpoint embedding, and contextual segmentation. To further improve the partition step, Landrieu and Boussaha proposed a supervised framework to oversegment a point cloud into pure superpoints [81]. This problem is formulated as a deep metric learning problem structured by an adjacency graph. In addition, a graph-structured contrastive loss is also proposed to help the recognition of borders between objects.

Another milestone in graph-based methods is DGCNN [82], which constructs a local neighborhood graph to extract the local geometric features and applies Convlike operations, named EdgeConv. An EdgeConv acts on graphs dynamically computed in each layer of the network and captures local geometric structure while maintaining permutation invariance.

### 5.6. Transformer-Based Methods

**Figure 13:**
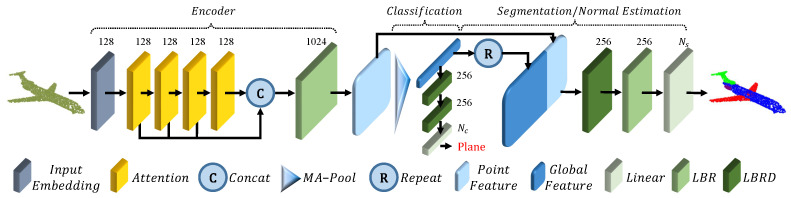
Point cloud transformer network architecture [83].

A very successful architecture presented recently in the literature is the transformer [84]. This architecture achieves a state-of-the-art performance in natural language processing tasks and is being employed also for image processing with good results. This type of model also lends itself well to point cloud processing because it is naturally independent of the input order. In [83], the authors propose Point Cloud Transformer (PCT) (Figure 13), which exploits the effectiveness of transformers for point cloud classification, normal estimation, semantic segmentation, and part segmentation, proposing some improvements with respect to the original architecture, in order to adapt it to the new domain.

In particular, the input embedding module is substituted with a neighbor embedding that, instead of obtaining point-wise features, aggregates them using Farthest Point Sampling (FPS) and K-Nearest Neighbors as in PointNet++ [50] in such a way that the receptive field is enlarged. On the other hand, the self-attention module usually computes:

(6) $$F_{out}=LBR\left(F_{sa}\right)-F_{in}$$

where *LBR* is a linear layer followed by batch normalization and ReLU activation function; $F_{sa}$ is the product between the attention vector and the values vector, and $F_{in}$ is the input to the layer. In this work, this layer is modified so that:

(7) $$F_{out}=LBR\left(F_{sa}-F_{in}\right)-F_{in}$$

similarly to a Laplacian operator in graph signal processing.

These improvements allow one to extract more meaningful features in the context of PCs, with respect to the standard transformer, allowing one to achieve a state-of-the-art performance in semantic segmentation and other meaningful tasks.

### 5.7. Performance Comparison between Different Approaches

In this section, we finally propose a quick comparison among some of the main aforementioned approaches for PCSS. In order to provide a fair comparison, we take into account the **Intersection over Union (IoU)** value, also referred to as the *Jaccard index*, which is one of the most commonly used metrics in semantic segmentation. It is defined as:

(8) $$\mathrm{IoU}=J\left(A,B\right)=\frac{\left|A\cap B\right|}{\left|A\cup B\right|}$$

where *A* and *B* denote the ground truth and the predicted segmentation maps, respectively; IoU ranges from 0 to 1. In general, the IoU is averaged over all the classes, i.e., the **Mean-IoU (mIoU)** is used.

**Figure 14:**
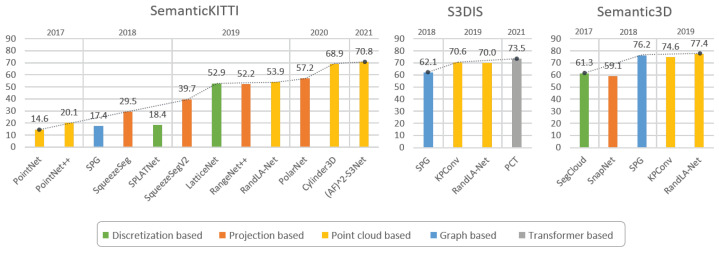
Graphical comparison of the main approaches for Point Cloud Semantic Segmentation in terms of mIoU percentage, on SemanticKITTI [35], S3DIS [44], and Semantic3D [33] datasets.

Table 2 presents some of the architectures mentioned, reporting their publication year, summarizing the methods used for such models and the results achieved on some popular datasets discussed in Section 3. These achievements are also visualized in Figure 14, where we underline for each approach the main broad category to which it belongs.

## 6. Compression

Point cloud compression is a very hot topic in 3D computer vision due to the increasing hype that self-driving cars and visors are arising. The former can acquire billions of points per day, while the latter is not capable of storing large amounts of information thus requiring an efficient way to store and exchange data compactly while retaining high reconstruction quality.

The basic approaches that tackle this problem employ clever data structures carefully designed for the task. One of the most effective ones is the octree, used for example in the MPEG compliant codec G-PCC [85]. It is based on a recursive partitioning of the space in octants and is represented as a tree. Octrees address sparsity by retaining information in the grid only where points are located. Usually, the coding tree derived from this procedure can be represented by 8-bit strings that are then encoded using standard lossless techniques (e.g., Huffman source coding). Octrees are used as the foundation for many other compression approaches [85,86] due to the high availability of software where they are implemented (for example the Point Cloud Library [87]). One of these approaches [88] exploits a tree-structured conditional entropy model to reduce the redundancy in the octree representation generated by LiDAR sensors in self-driving cars.

Other approaches exploit Graph Signal Processing (GSP), and specifically the Graph Fourier Transform, to capture the local structure of the 3D model [89,90]. These graphs are usually built either using KNN or by connecting with an edge all points that are closer than a certain threshold. In [91], the PC is encoded in two layers, the Base Layer (BL), i.e., where losslessly encodes a coarse representation of the 3D model, and the Enhanced Layer (EL), where finer-grained details are coded in a lossy manner using GSP. In particular, the residuals between the real model and the upsampled BL are multiplied by the basis (eigenvectors relative to the biggest eigenvalues of the Laplacian matrix of the local graph), thus obtaining some energy efficient coefficients that can be quantized, entropy coded, and transmitted or stored.

Finally, due to the success obtained by deep Autoencoders (AEs) in image compression [92,93,94,95,96,97], deep learning approaches have started to emerge for PCs compression also thanks to the advent of PCs specific networks such as [49]. In learned point cloud coding, approaches can be grouped into two main classes. In the first one, the data are handled as an unordered set of points (usually, and the adopted core architecture is PointNet [49]). In the second one, the PC is quantized in a grid and processed by convolutional layers.

Most of the point-based works are not strictly concerned with dimensionality reduction and reconstruction tasks and aim at training a neural model with efficient generative capabilities. As a matter of fact, autoencoders can be used for this purpose especially when the latent space is regularized, such as in Variational AE [98] or in Adversarial AE [99]. Moreover, most of these solutions mainly focus on the point cloud geometry (skipping the compression of their attributes, such as color components or normals). Notice that, even if the main focus of these methods is not strictly the compression of point cloud data, the efficiency of the related approaches makes them worthy of investigation and discussion.

### 6.1. Point-Set Autoencoders

In this section, PointNet-based architectures are overviewed [49]. PointNet is usually employed in architectures such as the encoder module. This allows one to have architectures invariant to input permutation, since the features are computed point-wise and then aggregated using a symmetric function. The latter technique is not easily extendable to the decoder; therefore, a simple fully connected architecture is usually adopted, which has to cope with the disadvantage of having the number of output points fixed.

The main weakness of this class of mechanisms is that both PointNet and fully connected networks are not really good at exploiting spatial correlation so it might take longer to learn meaningful compressed representations; moreover, MLP units are not reusing weights and require training a lot of parameters.

#### 6.1.1. Learning Representations and Generative Models for 3D Point Clouds

The first of these techniques is the one proposed by Achlioptas et al. in [100] where Autoencoders (AE), Generative Adversarial Networks (GANs), and Gaussian Mixture Models (GMMs) are explored as generative models for point cloud data. More precisely, the paper introduces a new PointNet-based AE architecture and evaluates different metrics as reconstruction objectives or performance evaluators for the generated samples. The AE processes PCs with fixed size of 2048 points, obtained by sampling points from shapes found in the ShapeNet and ModelNet datasets. Both EMD and CD are used as structural losses, and the results show a low reconstruction error (EMD≈0.043, $CD\approx 4.5\times 10^{-4}$ with 128 units in the hidden layer) on raw PC data. The remaining contributions of the paper are not discussed here as they involve generative models and not the main focus of this section, i.e., PC compression and reconstruction.

#### 6.1.2. Adversarial Autoencoders for Compact Representations of 3D Point Clouds

An improvement to [100] was proposed by Zamorski et al. in [101] by adding regularization to the latent space using an adversarial component trained with the Wasserstein criterion [102]. This discriminator is used to force a prior distribution on the latent space enabling the model to compactly represent point cloud data either with continuous or binary values. The model is designed to perform point cloud compression, clustering, and generation. The general encoder/decoder architectures and the considered datasets are the same as above. The neural network is trained in an end-to-end fashion using EMD loss.

#### 6.1.3. Folding Net

**Figure 15:**
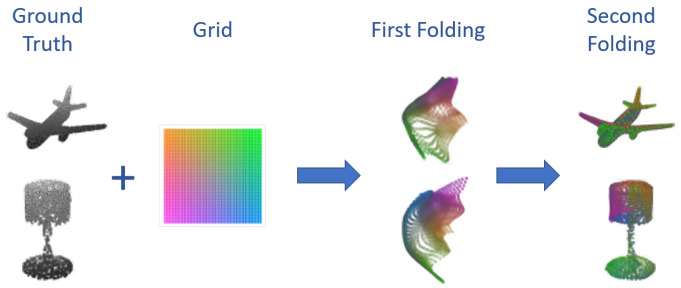
Examples of PCs and their reconstruction procedure.

A different workflow is presented in [103] where a new decoding technique is proposed. The approach followed by Yang et al. uses the features extracted by the encoder to fold a 2D grid in the shape of the object to reconstruct. This is performed in two steps: first, the latent space vector is concatenated with points in a 2D grid and processed by a 1D convolution similarly to PointNet to obtain an intermediate folding. This is then concatenated again with the former and processed to obtain the final result. Some examples of PC, intermediate foldings, and reconstructions can be seen in Figure 15.

In addition, in this case, ShapeNet is used as the training set, and the model is tested on ModelNet obtaining a final Chamfer distance of ≈0.03.

### 6.2. Convolutional Autoencoders

Like in their image-based counterparts, convolutional autoencoders have been widely exploited on voxel-based point cloud data. The current subsection overviews their main applications.

#### 6.2.1. Syndrome-Based Autoencoder

In the work [104], the proposed solution combines a classical CNN workflow with some ideas from Distributed Source Coding (DSC). In the DSC framework, the main idea is that the decoder already knows a code-word, called side information, highly correlated to the one that one would like to reconstruct. This implies that only a few correction bits, called syndromes, are sent in order to correct it.

The main idea here is that the receiver is sent a low-resolution version of the PC, encoded in a lossless manner and the syndromes computed by the encoder. This low-res model is then upsampled leading to a model with a lot of artifacts and bad quality. Then the decoder using the syndromes and the side information is able to remove these artifacts and reconstruct a point cloud similar to the original one.

The architecture used in this work is inspired by U-Net [105], but the features concatenated with the ones produced by the decoder are computed at the receiver side from the side information so that they do not need to be transmitted. In order to address the sparsity problem, the whole grid is divided into smaller blocks with dimensions 8×8×8, and only those that contain some useful information are processed by the network. The drawback of this approach is that it only exploits correlation inside the blocks. The main advantages are that the model can be trained with fewer data since it does not need to learn to encode complex shapes but just 8×8×8 cubes, it is highly parallelizable, and has small memory requirements as each cube is encoded and decoded independently.

An additional component is added to the loss function given that most of the reconstructed blocks are planar. This metric computes how similar the fitted planes in the original and reconstructed blocks are.

The dataset used to train and test the procedure is composed of 3D models provided by MPEG (see Section 3), this contains more complex samples w.r.t. ModelNet and ShapeNet since some of the models are acquired with structure from motion, stereo systems, or LiDAR and are thus not sampled from perfect synthetic meshes.

**Figure 16:**
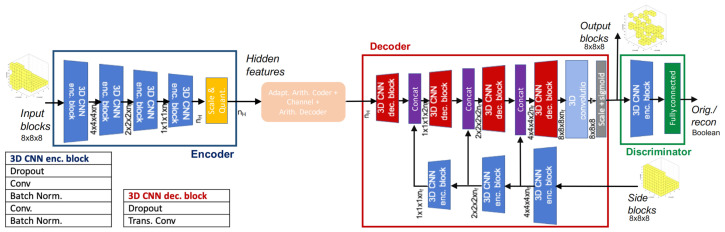
Scheme of the architecture proposed in [106].

An improvement is then introduced by the same author in [106] where a discriminator component is introduced after the decoder to distinguish between reconstructed and real data in order to improve the perceptual quality of the decoded blocks. The architecture proposed by this method can be seen in Figure 16.

#### 6.2.2. Learned-PCGC

Another work that follows a similar approach is [107]. The authors propose a compression framework that could be easily implemented on embedded systems due to the highly parallelizable procedure and the low amount of weights needed for the deep learning models.

The point cloud is first preprocessed by applying voxelization, and coordinates are downscaled, i.e., divided by a scale factor s>1 and rounded to the closest integer. Then, the grid is partitioned into blocks of dimension W×W×W. In addition, in this case, since the whole PC is processed in blockwise order, the coding performance is high, even with a relatively small model.

**Figure 17:**
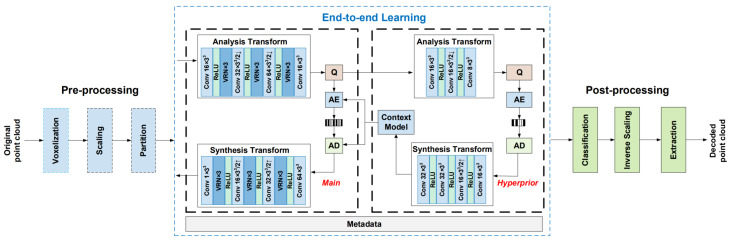
Architecture proposed in [107].

DL hyperprior coding [95] is adopted (see Figure 17) in order to improve the effect of entropy coding and thus obtain better compression rates. It consists of adding a side NN that is trained to predict the best parameter values for entropy coding leading to a better regularization and higher compression ratios.

At the decoder side, the PC is reconstructed by inverting all the aforementioned operations. The framework was trained on Shapenet and tested on the MPEG dataset achieving superior compression and quality w.r.t. to the G-PCC codec [85], i.e., the standard static point cloud coder developed by the MPEG group using non deep learning based techniques.

#### 6.2.3. PCGAE, Implicit/Explicit Quantization, and DL-PCSC

**Figure 18:**
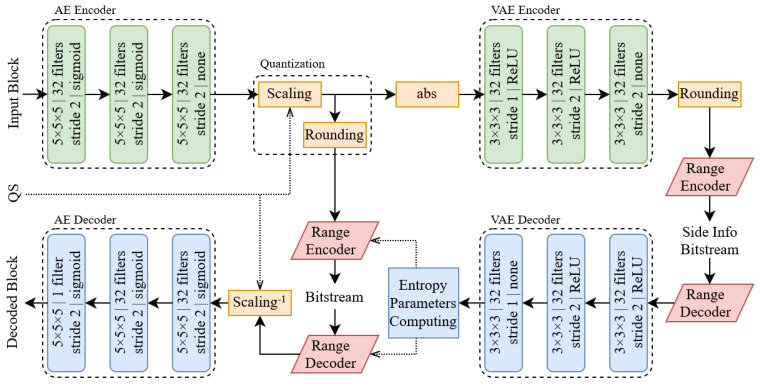
Architecture proposed in [108].

Other approaches were presented by Guarda et al. in [108]. Initially, the authors proposed in [109] a simple convolutional network where the latent space is quantized in order to perform entropy coding. Later in [108], the approach was improved by implementing the hyperpriors technique [95] and by proposing an implicit/explicit quantization framework (see Figure 18). In implicit quantization, a deep learning model is optimized to minimize a given rate–distortion tradeoff while, in the explicit one, the latent space is quantized with different step values depending on the required quality/rate tradeoff. This is performed to address an issue present in all the aforementioned works, i.e., multiple neural networks need to be trained in order to achieve different compression rates. With this type of approach, the overall number of networks that need to be trained is greatly reduced. To further refine this idea, ref. [110] was proposed; here, the latent space produced by the encoder is split into NL subsets of features that can be progressively encoded to obtain NL different quality levels. At the decoder side, the values of the missing levels are padded with zeroes leading to a loss in reconstruction quality.

#### 6.2.4. Brief Comparison between the Aforementioned Approaches

**Figure 19:**
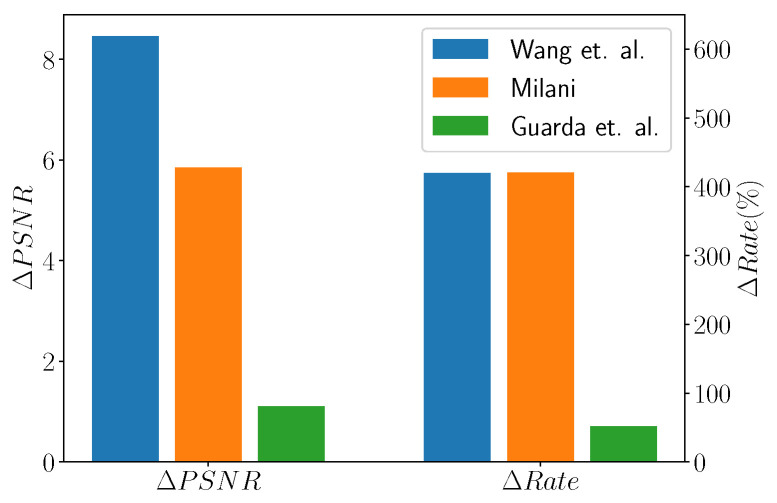
Bjontegard metrics for Wang et al. [107], Milani [106], and Guarda et al. [110] against TMC13.

When considering the works by Wang et al. [107], Milani [106], and Guarda et al. [110], it is possible to see from Figure 19 that the former achieves the best Bjontegaard improvement rates. Moreover, ref. [106] provides similar results with a simpler network that requires less training time. The approach by Guarda et al. gives the lowest reconstruction quality, but this is due to the fact that a single network is required to perform compression at different resolutions; therefore, this is the most flexible and the one with the lowest memory requirements among the three approaches.

## 7. Point Cloud Completion

Point cloud completion is the task of inferring the overall shape of an object given a partial observation. It is very common that real-world 3D data are incomplete; for example, models acquired by sensors installed on self-driving cars are usually sparse or incomplete.

PC completion approaches can be broadly summarized in the following categories:

- **Geometry methods**: the shapes are reconstructed from the partial input using interpolation [111,112,113,114], without the need for external data;
- **Symmetry methods**: symmetries and repeating patterns in the objects are detected [115,116,117,118] and used to reconstruct the missing parts;
- **Alignment methods**: either the partial input is matched and substituted with a shape in a database [119,120,121,122], or multiple parts are matched and merged together [123,124,125] in order to obtain the full surface;
- **Learned methods**: a model learns a probabilistic representation of the possible shapes, and then it produces the most likely output that might have generated the input it was fed [126,127].

When considering learned methods, neural networks are generally the most performing solutions. There is great parallelism between compression and completion because also in this case the model requires one to extract meaningful features from the input and to reconstruct the original shape. Consequently, also in PC completion the most successful architectures are AEs either with a PointNet-based encoder (CD or EMD losses) or with a voxelized representation of the PC using CNNs and MSE.

### 7.1. Point Completion Network

**Figure 20:**
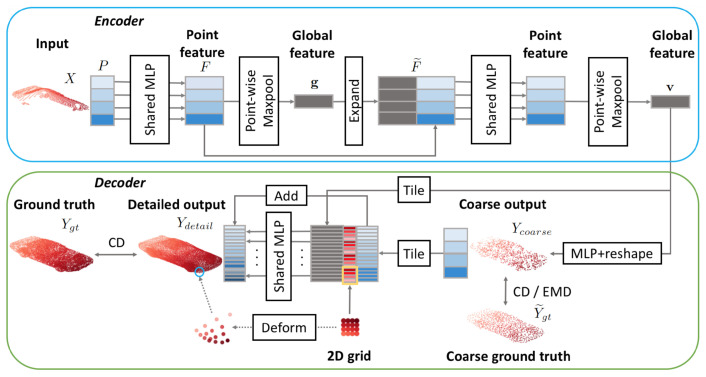
Point Completion Network [128] architecture.

Great results on this matter were obtained by Yuan et al. [128] (see Figure 20), where the authors combined the techniques proposed in [100] and in [103] (see Section 6.1) in order to achieve better reconstruction performance. This was motivated by the fact that they noticed that the fully connected decoder is better at reconstructing a low-density version of the object, while the grid deformation procedure is better at distributing the points along the surface.

The encoder applies PointNet twice: the first time, a matrix of features *F* is computed and then the global representation is obtained as g=maxpool(F). Then, in the second step, *g* is concatenated to each feature vector in *F*, and they are processed again by a PointNet-like architecture to obtain the final result. The decoder, on the other hand, starts with some fully connected layers, used to predict a low-resolution version of the object that should be reconstructed. Then, in the second stage, each predicted point is combined with a 2D grid that with the folding operation turns into a patch. Finally, all these are merged together to obtain the full PC.

The overall loss is obtained by combining a Chamfer distance component between the reconstruction and the ground truth, with the earth moving distance between the coarse reconstruction and a downsampled version of the expected PC. The dataset used for training is ShapeNet, and the partial inputs are generated by backprojecting 2.5D images into 3D to obtain a result that resembles data acquired by real sensors.

### 7.2. Point Fractal Network

**Figure 21:**
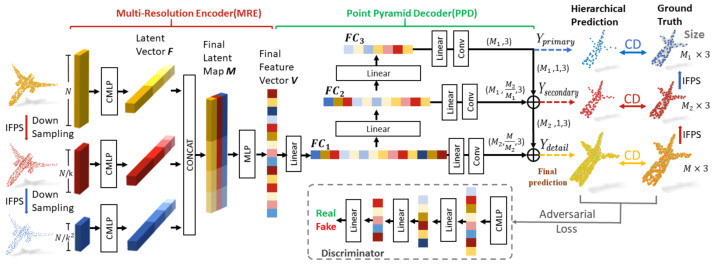
Point Fractal Network [129] architecture.

The work by Huang et al. [129] uses a PointNet-based encoder called Combined Multilayer Perceptron (CMLP) (see Figure 21). Unlike [128], CMLP applies a max-pooling on the last three layers (instead of the last one only). The feature representations are aggregated into a vector that contains both global and local information. This procedure is applied three times, first on the partial point cloud and then on two subsampled versions of the latter using the iterative farthest point sampling algorithm. The three feature vectors are aggregated and processed by an MLP in order to compute the latent space representation of the PC.

On the decoder side, only the missing part is reconstructed. This means that the original geometry of the object is preserved. This is performed hierarchically, i.e., starting from the latent representation 3 different feature layers $FC_{1},FC_{2},FC_{3}$ which are produced by using a fully connected network. Each of these is responsible for predicting the missing part at a different resolution. This allows one to build the missing component in a hierarchical manner, which shows good improvements in the reconstruction quality.

The loss is composed of a multistage completion loss and an adversarial component. The former is computed by aggregating the Chamfer Distance between the reconstruction at the different resolutions and the ground truth together with its downsampled versions, while the latter is obtained by adding a discriminator that has to understand if the missing part is real or reconstructed.

### 7.3. 3D Point Capsule Networks

**Figure 22:**
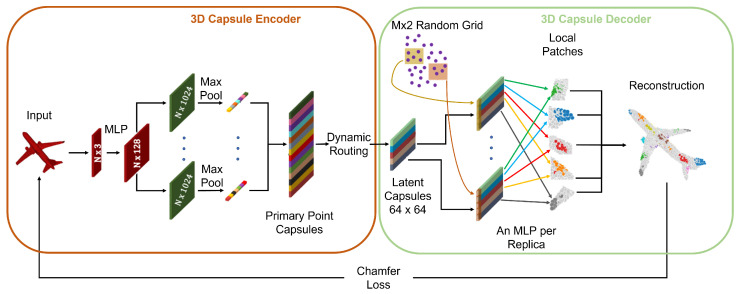
3D Point Capsule Network [130] architecture.

Many of the techniques used for PC processing are extensions of successful ideas for 2D multimedia data. One example is the work by Zhao et al. [130] where capsule networks [131] and the dynamic routing algorithm [132] are adapted to 3D data (see Figure 22) in order to learn more meaningful features in the latent space of the autoencoder. This is made possible by the high representative power of capsules whose output is a vector, where the norm represents the probability that the feature encoded in the vector is present in the input, while the direction represents the actual feature.

The encoder is built by extracting per-point features using the same technique as in PointNet, and then these are fed into multiple independent convolutional layers followed by max-pooling. The outputs are concatenated into a vector called primary point capsules, and these are then clustered into higher level latent capsules using the dynamic routing algorithm. The capsules are concatenated with random grids, following a procedure similar to [103]: the grids are deformed accordingly to capsule features and patched together to obtain the final reconstructed model. In addition, in this case, the dataset used for training is ShapeNet, and the reconstruction loss is the Chamfer distance.

### 7.4. GRNet

One of the most successful architectures for point cloud completion is GRNet [133]. Here, two brand-new differentiable layers were proposed: *Gridding* and *Gridding reverse*. Their purpose is to transform the PCs into grids that can be processed by convolutional layers without adding quantization noise. Optimizing the parameters using Chamfer Loss usually results in predictions that average the various possible modes of the output [134]. As a consequence, a new Gridding loss is proposed, i.e., the L1 distance between *Gridding*($y_{pred}$) and *Gridding*($y_{true}$). The *Gridding* operation computes a grid around the model, assigning a value $w_{i}$ to each vertex $v_{i}=\left(x_{i}^{v},y_{i}^{v},z_{i}^{v}\right)$ of the grid. In order to retain information about the points in the various cells of the grid, $w_{i}$ is computed as:

(9) $$w_{i}=\sum_{p\in N(v_{i})}\frac{w(v_{i},p)}{\left|N(v_{i})\right|}$$

where $N(v_{i})$ is the set of points of the PC lying in the eight cells that are adjacent to $v_{i}$, and the interpolation function $w(v_{i},p)$ is defined as:

(10) $$w\left(v_{i},p\right)=\left(1-\right|x_{i}^{v}-x|)(1-|y_{i}^{v}-y|)(1-|z_{i}^{v}-z\left|\right)$$

The *Gridding reverse* operation on the other hand generates one point $p_{i}^{c}$ for each cell in the grid as:

(11) $$p_{i}^{c}=\frac{\sum_{\theta \in \Theta^{i}}w_{\theta }^{\prime }v_{\theta }}{\sum_{\theta \in \Theta^{i}}w_{\theta }^{\prime }}$$

where $\Theta^{i}$ is the set of the eight vertices around the ith cell; in general, no point is generated if $\sum_{\theta \in \Theta^{i}}w_{\theta }^{\prime }=0$. The network operates in five steps:

1. The incomplete PC is transformed in a grid using the *Gridding* operation;
2. The grid is processed by a CNN with skip connections in a U-Net fashion, and this step should reconstruct the missing part of the input PC;
3. *Gridding reverse* is used to produce a coarse point cloud $P^{c}$;
4. The next step is cubic feature sampling. Here, 2048 points are sampled from the coarse point cloud. For each point, the features computed by the first three transposed convolution layers (relative to the eight vertices around the cell where the point lies) are concatenated together to generate a big feature vector $F^{c}$;
5. A multilayer perceptron processes these features to predict the residual offset between the point in the coarse and the final completed PC. Then, the final PC is computed as $P=MLP\left(F^{c}\right)+Tile\left(P^{c},r\right)$ where the Tile operation repeats the points $P^{c}$, *r* times, so that the final PC has 2048r points.

As previously mentioned, the loss is computed as the $L_{1}$ distance between the grid produced by the ground truth and the one produced by the prediction:

(12) $$L_{gridding}=\frac{1}{N_{G}^{3}}||Gridding\left(P_{rec}\right)-Gridding\left(P_{true}\right){\left|\right|}_{1}$$

where $N_{G}$ is the resolution of the grid.

The training set was derived from ShapeNet similarly to [128] while the Completion3D benchmark [135] and the KITTI [34] datasets were used for performance evaluation.

### 7.5. Other Strategies

Other noteworthy deep-learning-based approaches are AtlasNet and TopNet.

AtlasNet [136] is one of the first approaches for PC completion. It inspired the Point Completion Network work [128], and in fact, it works in a similar way: a coarse reconstruction of the PC is computed, and randomly sampled 2D grids are folded around each point.

Otherwise, in TopNet [135], the decoder reconstructs the whole PC in a hierarchical rooted tree structure. This is performed by using *n* MLPs to predict *n* new features for the next layer of the tree. These features are then concatenated with the latent space vector, and the same procedure is repeated. In the end, the final layer predicts points that are aggregated to produce the PC.

## 8. Conclusions

This survey presents an updated overview of the main research trends in deep point cloud processing. The paper highlights the newest research trends and proposes a new taxonomical organization that includes some of the main peculiarities of PC structures and the adopted neural architectures. The current state-of-the-art PC technology focuses on semantic scene understanding, coding, and completion tasks: the current paper proposes detailed highlights on these aspects, while including some insights on other related operations. Among them, we can identify semantic segmentation, classification and object detection, generative reconstruction algorithms, and upsampling (deeply connected with compression and completion). Such tasks usually exploit very similar architectures and training procedures, applicable in a variety of different scenarios, which are thereby reviewed in this paper via highlighting their differences and similarities. Special attention was also devoted to the PC acquisition mechanism, which has recently proved to be crucial in characterizing the statistics of the processed data. Being that DL solutions are highly data oriented, the peculiarities of the acquiring technologies strongly affect the dataset and its quality. As a matter of fact, this overview also poses attention on the available datasets and on their uses for the different tasks.

Starting from these premises, the analysis highlights that there is still plenty of room for improvements, and future research trends can be summarized as follows.

- **Semantic Scene Understanding**: as it is a widely explored field, many solutions developed are able to provide accurate results. Point-based methods are the actual direction of research for their lossless property, and the main hot topic is the development of networks that include the temporal dimension, as only a few works already exist. Moreover, given the huge computational capabilities required by these tasks, modifications to the learning procedures are being investigated, and future works should point to this direction, particularly aiming at faster and lighter solutions.
- **Compression**: right now only geometry coding for static PCs has been addressed; therefore, future research works may tackle attributes (color, normals, etc.), dynamic point clouds, and also the study of techniques for better rate control. Moreover, since some applications target the PC visualization, future compression strategies will be dealing with PC rendering and interpolation as well (see [21] as an example).
- **Completion**: point cloud completion networks already achieve very good reconstruction results. To the best of our knowledge, the main issue is that most of these approaches require very big and computationally complex networks which might be unsuitable for real-time applications and small devices with low computational capabilities. Future works should try to provide results comparable to the state of the art but with smaller computational time, memory requirements, and energy consumption. Moreover, the last year has witnessed a rising hype towards the Neural Radiance Field (NeRF) [137] for 3D data visualization and interpolation. Although these solutions have been adopted so far on light-field imaging and multi-view rendering, their application to SfM point clouds comes as a natural extension.
- **Generative approaches**: when considering generative methods for PC coding, these are still in very early stages; in particular, mostly single 3D models have been considered, and moving toward automatic PC scene generation might be a very promising research direction in the field of PC generation.

Overall, point cloud processing is now moving towards the direction of new technologies driven by the latest development of deep learning and GPUs. However, research is moving also towards the exploration of new learning techniques and methodologies, e.g., Continual Learning, Contrastive Learning, Coarse-to-Fine Learning, and Domain Adaptation techniques, to adapt the models to data and improve them even in the absence of huge computational devices.

## Figures and Tables

**Table 1:** Structure of the paper and related references.

Sections	Related References
(1) Introduction	[1,2,3,4,5,6,7,8,9,10,11,12,13,14,15,16,17,18,19,20,21,22,23,24,25,26,27,28,29,30,31]
(2) Point Clouds as Data Structures	
(2.1) Point Cloud Data	[32]
(2.2) Acquisition Systems	[25,27,28,33,34,35]
(2.3) Other Data Structures	[4]
(3) Datasets	
(3.1) ShapeNet	[36,37]
(3.2) ModelNet	[38]
(3.3) MPEG	[39]
(3.4) 8i Voxelized Full Bodies	[40]
(3.5) Stanford 3D Indoor Scene Dataset	[41,42]
(3.6) KITTI	[43]
(3.7) SemanticKITTI	[44]
(3.8) SynthCity	[45]
(3.9) Other Recent LiDAR Datasets for Automotive Applications	[46,47,48]
(4) General Purpose Deep Learning Techniques	
(4.1) Architectures	[29,30,49,50,51]
(4.2) Losses	[52]
(5) Semantic Scene Understanding	
(5.1) Disambiguation	
(5.2) Discretization-Based Models	[30,38,51,53,54,55,56,57,58,59,60]
(5.3) Projection-Based Models	[61,62,63,64,65,66,67,68,69,70,71,72]
(5.4) Point Clouds-Based Models	[6,32,34,49,50,73,74,75,76,77,78,79]
(5.5) Graph-Based Methods	[80,81,82]
(5.6) Transformer-Based Methods	[50,83,84]
(5.7) Performance Comparison between Different Approaches	
(6) Compression	[49,85,86,87,88,89,90,91,92,93,94,95,96,97,98,99]
(6.1) Point-Set Autoencoders	[49,100,101,102,103]
(6.2) Convolutional Autoencoders	[85,95,104,105,106,107,108,109,110]
(7) Point Cloud Completion	[111,112,113,114,115,116,117,118,119,120,121,122,123,124,125,126,127]
(7.1) Point Completion Network	[100,103,128]
(7.2) Point Fractal Network	[128,129]
(7.3) 3D Point Capsules Networks	[103,130,131,132]
(7.4) GRNet	[43,128,133,134,135]
(7.5) Other strategies	[128,135,136]
(8) Conclusions	[21,137]

**Table 2:** Comparison of some of the main approaches for Point Cloud Semantic Segmentation in terms of mIoU percentage, on SemanticKITTI [35], S3DIS [44], and Semantic3D [33] datasets.

	Year	SemanticKITTI	S3DIS	Semantic3D	Category
PointNet [49]	2017	14.6	-	-	PC (MLP)
PointNet++ [50]	2017	20.1	-	-	PC (MLP)
SegCloud [54]	2017	-	-	61.3	Disc (D)
SnapNet [67]	2018	-	-	59.1	Proj (MV)
SqueezeSeg [68]	2018	29.5	-	-	Proj (Sph)
SPGraph [80]	2018	17.4	62.1	76.2	Graph
SPLATNet [58]	2018	18.4	-	-	Disc (S)
SqueezeSegV2 [70]	2019	39.7	-	-	Proj (Sph)
LatticeNet [59]	2019	52.9	-	-	Disc (S)
KPConv [74]	2019	-	70.6	74.6	PC (C-Conv)
RangeNet++ [71]	2019	52.2	-	-	Proj (Sph)
RandLA-Net [60]	2019	53.9	70.0	**77.4**	PC (MLP)
PolarNet [72]	2020	57.2	-	-	Proj (Cyl)
Cylinder3D [77]	2020	68.9	-	-	Proj (Cyl) + PC (D-Conv)
PTC [83]	2021	-	**73.5**	-	Transformer
(AF)${}^{2}$-S3Net [75]	2021	**70.8**	-	-	Disc (D) + PC (C-Conv)

## Data Availability

All of the datasets mentioned in this paper were properly cited, and the download links are either specified in the bibliography or are present in the papers (also referenced in the bibliography) that explain how they were built and what type of data they contain.

## Abbreviations

The following abbreviations are used in this manuscript:

AR	Augmented Reality
BL	Base Layer
CD	Chamfer Distance
CDBN	Convolutional Deep Belief Network
CNN	Convolutional Neural Network
CRF	Conditional Random Field
DL	Deep Learning
DSC	Distributed Source Coding
EL	Enhanced Layer
EMD	Earth Moving Distance
FMCW	Frequency-Modulated Continuous Wave
GSP	Graph Signal Processing
GPU	Graphics Processing Units
KNN	K-Nearest Neighbors
IMU	Inertial Measurement Unit
LiDAR	Light Detection And Ranging
MLP	Multilayer Perceptron
mIoU	mean Intersection over Union
NN	Neural Network
PC	Point Cloud
SS	Semantic Segmentation
SC	Sparse Convolutions
UDA	Unsupervised Domain Adaptation
VR	Virtual Reality
